# Knockdown of protein interacting with C α kinase 1 aggravates sepsis-induced acute liver injury by regulating the TLR4/NF-κB pathway

**DOI:** 10.1038/s41598-023-38852-w

**Published:** 2023-07-24

**Authors:** Huijun Wang, Ting Ma, Qianqian Bao, Lijun Zhu, Tingting Ying, Yulong Yu

**Affiliations:** 1 Department of Anesthesia, Taizhou Hospital of Zhejiang Province Affiliated to Wenzhou Medical University, 150, Ximen Street, Linhai City, Taizhou, 317000 Zhejiang China; 2 Department of Anesthesia, The First Affiliated Hospital of Zhejiang Chinese Medical University (Zhejiang Provincial Hospital of Chinese Medicine), Hangzhou, 310000 Zhejiang China; 3 Department of Operating Room, Taizhou Hospital of Zhejiang Province Affiliated to Wenzhou Medical University, Taizhou, 317000 Zhejiang China

**Keywords:** Cell biology, Molecular biology, Medical research, Molecular medicine, Pathogenesis

## Abstract

Acute liver injury (ALI) may manifest at any phase of sepsis, yet an explicit therapeutic approach remains elusive. In this study, LPS and cecum ligation and puncture (CLP) were utilized to establish an inflammatory cell model and a murine model of sepsis-induced liver injury, respectively, aiming to explore the potential protective effect of protein interacting with C α kinase 1 (PICK1) on sepsis-induced ALI and its underlying mechanisms. In both the cell supernatant and the murine whole blood, the concentrations of inflammatory factors were quantified by ELISA, while the protein and mRNA expressions of PICK1, cleaved-PARP-1, caspase1, TLR4, IκBα, and NF-κB were assessed via western blot and qRT-PCR. The outcomes revealed that the knockdown of PICK1 increased the levels of inflammatory factors and apoptosis, alongside activation of TLR4/NF-κB signaling pathway-related factors in both in vivo and in vitro models. Moreover, the murine liver samples were subjected to Hematoxylin–Eosin (HE) staining for assessment of histopathological morphology. The HE staining and liver injury scoring results manifested a markedly exacerbated hepatic damage in PICK1 knockout mice as compared to WT mice following CLP. Furthermore, the liver macrophages were isolated from murine livers, and the expression and activity of the factors associated with the TLR4/NF-κB signaling pathway were verified through RT-qPCR and western blot, and EMSA assay demonstrated an augmented NF-κB activity subsequent to PICK1 knockout. Finally, the expression and localization of PICK1 in macrophages were further scrutinized via immunofluorescence, and the interaction between PICK1 and TLR4 was identified through co-immunoprecipitation. In conclusion, the knockdown of PICK1 appeared to modulate inflammatory factors by activating the TLR4/NF-κB signaling pathway, thereby exacerbating hepatic damage induced by sepsis.

## Introduction

Sepsis is an extensive systemic inflammatory reaction that can lead to multiple organ failure, shock, and death in severely ill individuals with infectious diseases^1, 2^. The liver, being the largest organ in the human body, serves various pivotal functions encompassing metabolism, detoxification, bile secretion, immune defense, and coagulation, rendering it one of the organs most frequently affected in the aftermath of sepsis^3^. Acute liver injury (ALI) may manifest at any phase of sepsis, and its metabolic and functional alterations exert an impact on the progression of sepsis^4^. Despite some advancements in the sepsis-induced ALI, its underlying pathogenesis is intricate and multifactorial, involving intricate interactions among various factors, and an explicit therapeutic approach is yet to be established^5^. Hence, it is imperative to explore more potent therapeutic agents and molecular targets to combat sepsis-induced ALI.

Protein interacting with C α kinase 1 (PICK1) is widely distributed in the body’s tissues and cells , comprising the coiled-coil region, acidic amino acid region, PDZ domain, and BAR domain. Previous investigations have elucidated that PICK1 can alleviate inflammatory pain and had a vital role in the development of various critical diseases^6–8^. Additionally, it has been demonstrated that PICK1 can protect against sepsis-induced lung injury by regulating glutathione (GSH) synthesis^9^, as well as prevent LPS-induced apoptosis of renal tubular epithelial cells by reducing excessive ROS levels^10^. Based on these findings, we hypothesize the existence of a protective mechanism involving PICK1 against sepsis-induced ALI.

Lipopolysaccharide (LPS) is widely acknowledged as the primary trigger of systemic inflammatory syndrome and is commonly employed as an inducer for cellular inflammation modeling^11^. It has been demonstrated that toll-like receptor 4 (TLR4) expressed on intrahepatic cell membranes can induce an inflammatory response in liver tissue by initiating downstream inflammatory gene expression and cytokine release^12^, and that aberrant TLR4 expression is associated with the development of diseases such as acute liver failure^13^. Nuclear Factor- Kappa B (NF-κB), a ubiquitous nuclear transcription factor, plays an essential pivotal role in sepsis^14^, as it can be activated by LPS for transcription to produce diverse cytokines such as tumor necrosis factor-α (TNF-α) and interleukin-1 (IL-1), and can further activate to expand inflammation between tissues and cells^15^. Xie et al. have confirmed that PICK1 exerts a protective effect on the liver by inhibiting the activity of NF-κB signaling pathway, which reduces the release of excessive inflammatory factors due to liver macrophages polarization in LPS-induced ALI^16^. Nevertheless, whether PICK1 can regulate the inflammatory response mediated by the TLR4/NF-κB pathway in sepsis and the underlying mechanisms involved have not yet been reported.

In this study, LPS and cecum ligation and puncture (CLP) were utilized to establish an inflammatory cell model and a murine model of sepsis-induced liver injury, respectively, to explore whether PICK1 can plays a protective role in sepsis-induced ALI by suppressing the TLR4/NF-κB pathway signaling through in vitro and in vivo experiments.

## Results

### PICK1 knockdown increases apoptosis and inflammation levels in cell models

**Figure 1:**
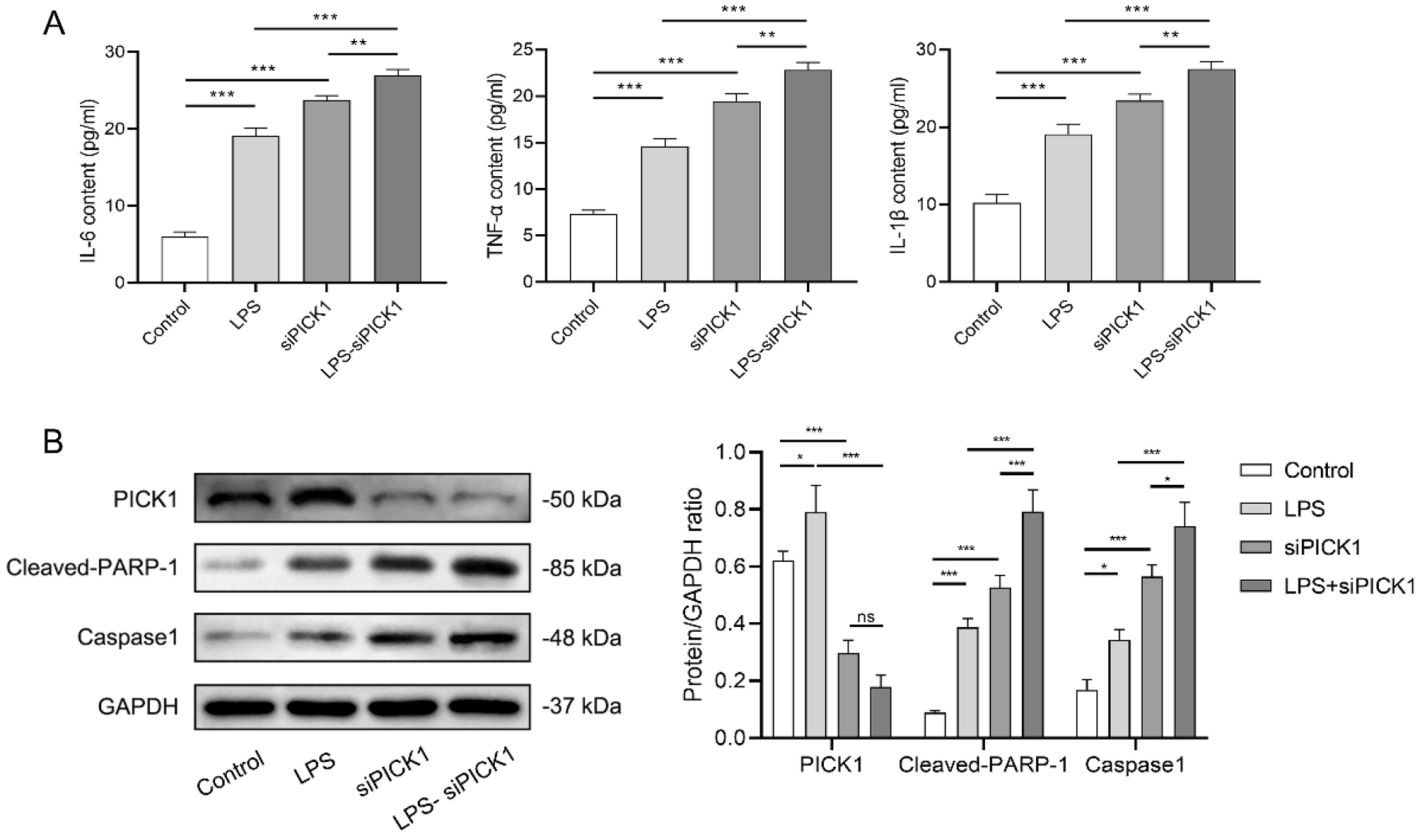
PICK1 knockdown increases apoptosis and inflammation levels in cell models. In the cell supernatant, (**A**) the concentration of inflammatory factors including IL-6, TNF-α, and IL-1β of each group were detected by ELISA; (**B**) The protein expression of PICK1, cleaved-PARP-1, and caspase1 of each group were detected by western blot. *p < 0.05, **p < 0.01, ***p < 0.001.

The results showed that the concentration of inflammatory factors in the LPS group was significantly higher compared to the control group (*p* < 0.001), whereas the LPS-siPICK1 group exhibited a notable further increase in inflammatory factors compared to the siPICK1 group (*p* < 0.01). Moreover, the concentration of inflammatory factors in both the control group and the LPS group was considerably increased after the knockdown of PICK1 (*p* < 0.001, Fig. 1A).

As shown in Fig. 1B, the PICK1 protein expression was considerably higher in the inflammatory cells than that in the controls (*p* < 0.05), and the significant reduction of PICK1 in the siPICK1 group indicated that the PICK1 was successfully knockdown (*p* < 0.001). Furthermore, there was a significant increase in the expression of cleaved PARP-1 and caspase1 in the LPS group compared to control cells (*p* < 0.05, *p* < 0.001), and the siPICK1 and LPS-siPICK1 groups exhibited even higher levels of cleaved PARP-1 and caspase1 expression (*p* < 0.001).

### PICK1 knockdown activates TLR4/NF-κB signaling pathway in cell model

**Figure 2:**
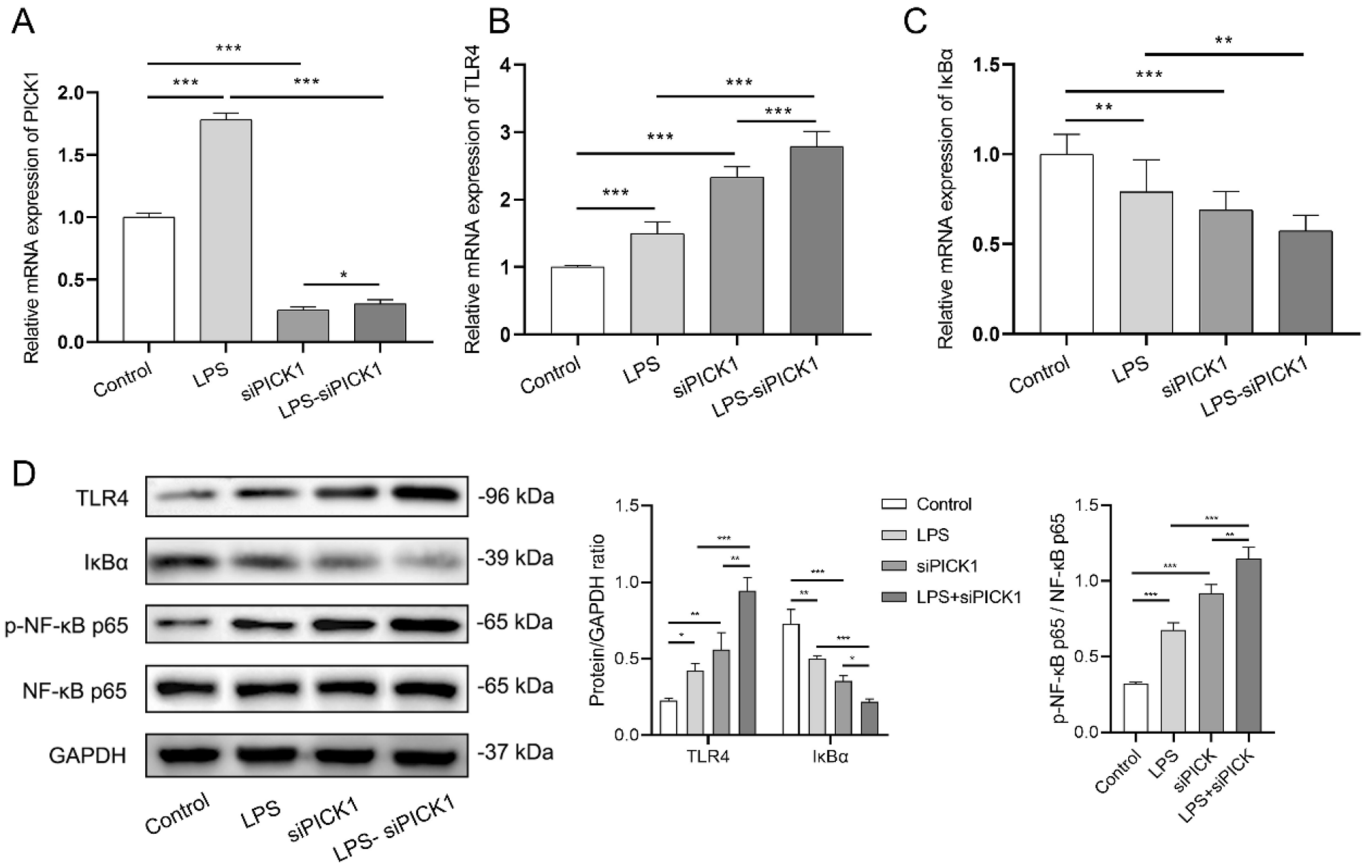
PICK1 knockdown activates TLR4/NF-κB signaling pathway in cell model. In the cell supernatant, the mRNA levels of (**A**) PICK1, (**B**) TLR4, and (**C**) IκBα of each group were detected by RT-PCR; (**D**) The protein expression of TLR4, IκBα, and the phosphorylation levels of the NF-κB p65 of each group were detected by western blot. *p < 0.05, **p < 0.01, ***p < 0.001.

In order to further investigate the underlying mechanism of PICK1 in inflammation, the mRNA levels of PICK, TLR4, and IκBα in the cell supernatant were examined by RT-PCR (Fig. 2A–C). The PICK1 and TLR4 mRNA levels in the LPS group were considerably higher than those in the control group (*p* < 0.001). Following PICK1 knockdown, both the control and LPS groups showed a significant increase in TLR4 mRNA levels (*p* < 0.001), while the mRNA expression trend of IκBα was found to be opposite to that of TLR4. As depicted in Fig. 2D, the protein expression of TLR4 and IκBα were consistent with the PCR findings. The IκBα protein expression was considerably decreased in the LPS group compared to the control group (*p* < 0.01), and it was further prominently lessened in both the siPICK1 and LPS-siPICK1 groups (*p* < 0.001). Since IκBα was a suppressor protein of NF-κB, these findings suggested that the knockdown of PICK1 allowed for further activation of NF-κB. Additionally, the phosphorylation level of NF-κB p65 was noticeably greater in the LPS group compared to the control group (*p* < 0.001), and further significantly increased in both the siPICK1 and LPS-siPICK1 groups (*p* < 0.001).

### PICK1 knockout increases apoptosis and activates TLR4/NF-κB inflammatory signaling pathway in animal models

**Figure 3:**
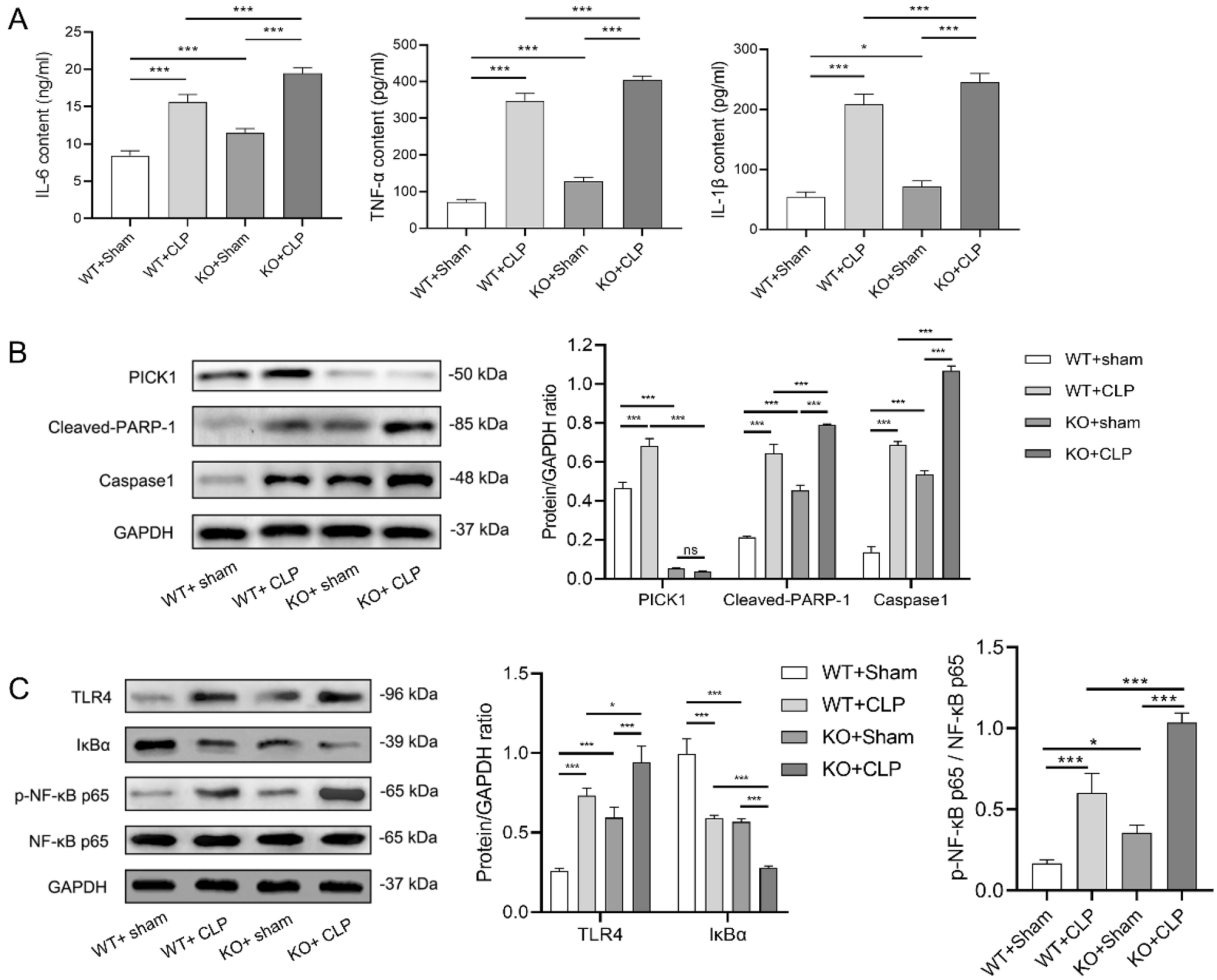
PICK1 knockout increases apoptosis and activates TLR4/NF-κB inflammatory signaling pathway in animal models. In the whole blood of mice, (**A**) the concentration of inflammatory factors including IL-6, TNF-α, and IL-1β of each group were detected by ELISA; (**B**) The protein expression of PICK1, cleaved-PARP-1, and caspase1 of each group were detected by western blot; (**C**) The protein expression of TLR4, IκBα, and the phosphorylation levels of the NF-κB p65 of each group were detected by western blot. *p < 0.05, ***p < 0.001.

Inflammatory factor content was found to be considerably greater in the CLP groups compared to the sham groups (*p* < 0.001), and significantly higher in the KO groups compared to the WT groups (*p* < 0.05, *p* < 0.001, Fig. 3A). As depicted in Fig. 3B, the PICK1 protein expression were noticeably higher in the CLP group in comparison to the sham group in wild-type mice (*p* < 0.001), and the apoptosis levels were also significantly greater in the KO + CLP group than in the KO + sham and the WT + CLP groups (*p* < 0.001). As presented in Fig. 3C, the TLR4 protein expression and the NF-κB p65 phosphorylation levels in the KO groups were noticeably higher than those in the WT groups (*p* < 0.05, *p* < 0.001), and it were noticeably elevated in the CLP groups compared to the sham groups (*p* < 0.001). Additionally, the protein expression of IκBα was significantly lower in the KO groups than the WT groups (*p* < 0.001) and considerably lower in the CLP groups than the sham groups (*p* < 0.001), respectively. This shows that the results of animal experiments were consistent with the results of cell experiments.

### PICK1 knockout exacerbates sepsis-induced liver injury

**Figure 4:**
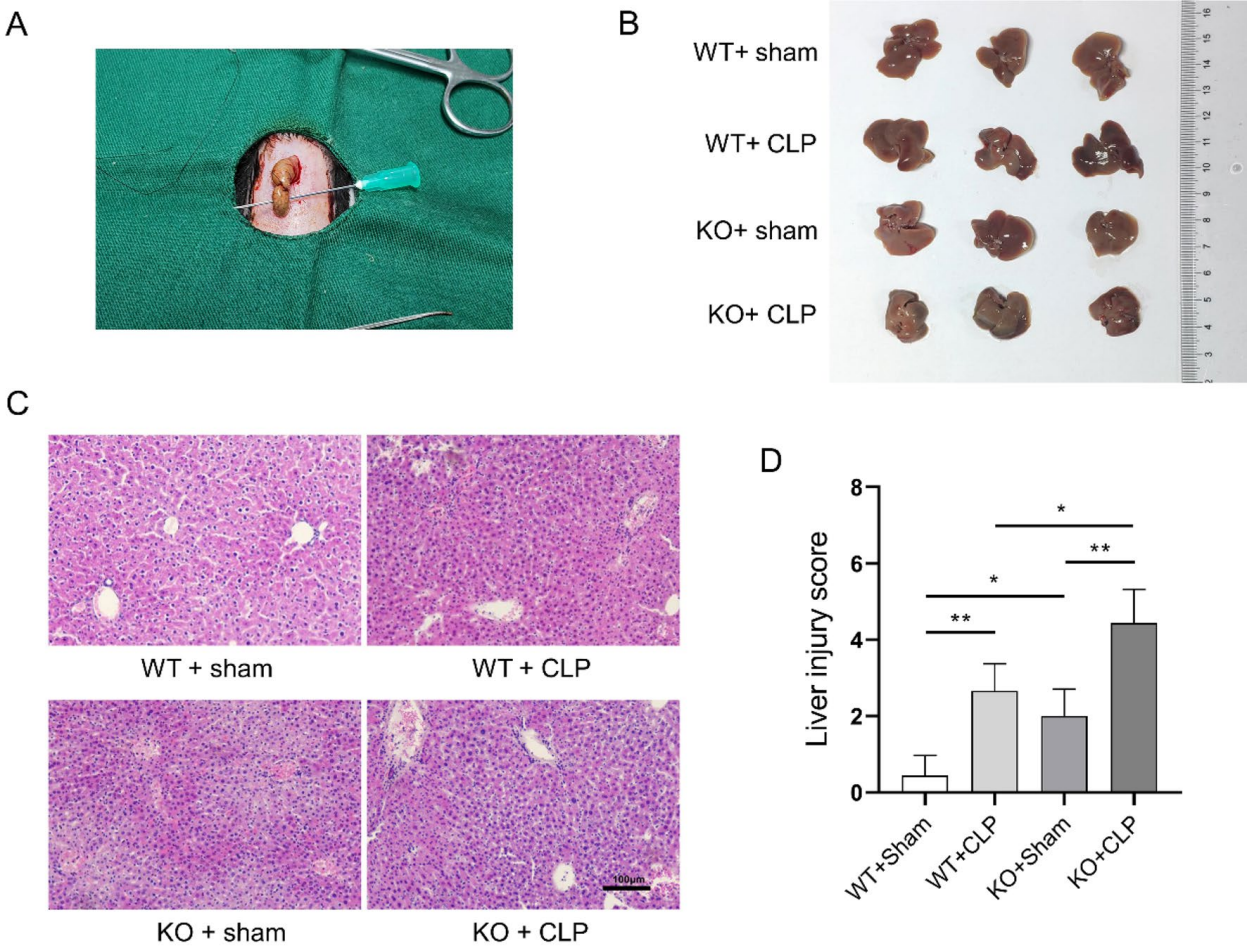
PICK1 knockout exacerbates sepsis-induced liver injury (n = 3). (**A**) The livers of the mice were removed, (**B**) the general changes of the hepatic organ were examined with unaided vision; (**C**) The histopathological morphology of the liver was evaluated using Hematoxylin–Eosin (HE) staining; (**D**) The liver injury score.

Upon removal of the livers of the mice, it was observed that that compared to the WT + sham group, the livers of the WT + CLP group and the KO + sham group were slightly swollen and congested, while the livers of the KO + CLP group were severely swollen and whitened (Fig. 4A,B). Subsequent HE staining revealed that the liver tissue of the WT + sham group exhibited neatly arranged liver cells, intact hepatic sinusoids, and cytoarchitecture, with no noticeable pathological changes observed. The WT + CLP group and the KO + sham group exhibited slight edema of hepatocytes, a few necrotic hepatocytes with a lighter degree than the KO + CLP group, a minor infiltration of inflammatory cells, with insignificant erythrocyte exudation. In contrast, the liver cells of the KO + CLP group were edematous, necrotic, hemorrhagic, and disorganized, with a substantial infiltration of inflammatory cells and a certain amount of red blood cell exudation (Fig. 4C). In addition, the liver injury scoring results revealed a significantly exacerbated liver damage in PICK1 knockout mice compared to WT mice following CLP (*P* < 0.01, Fig. 4D).

### PICK1 knockout activates TLR4/NF-κB signaling pathway in liver macrophages

**Figure 5:**
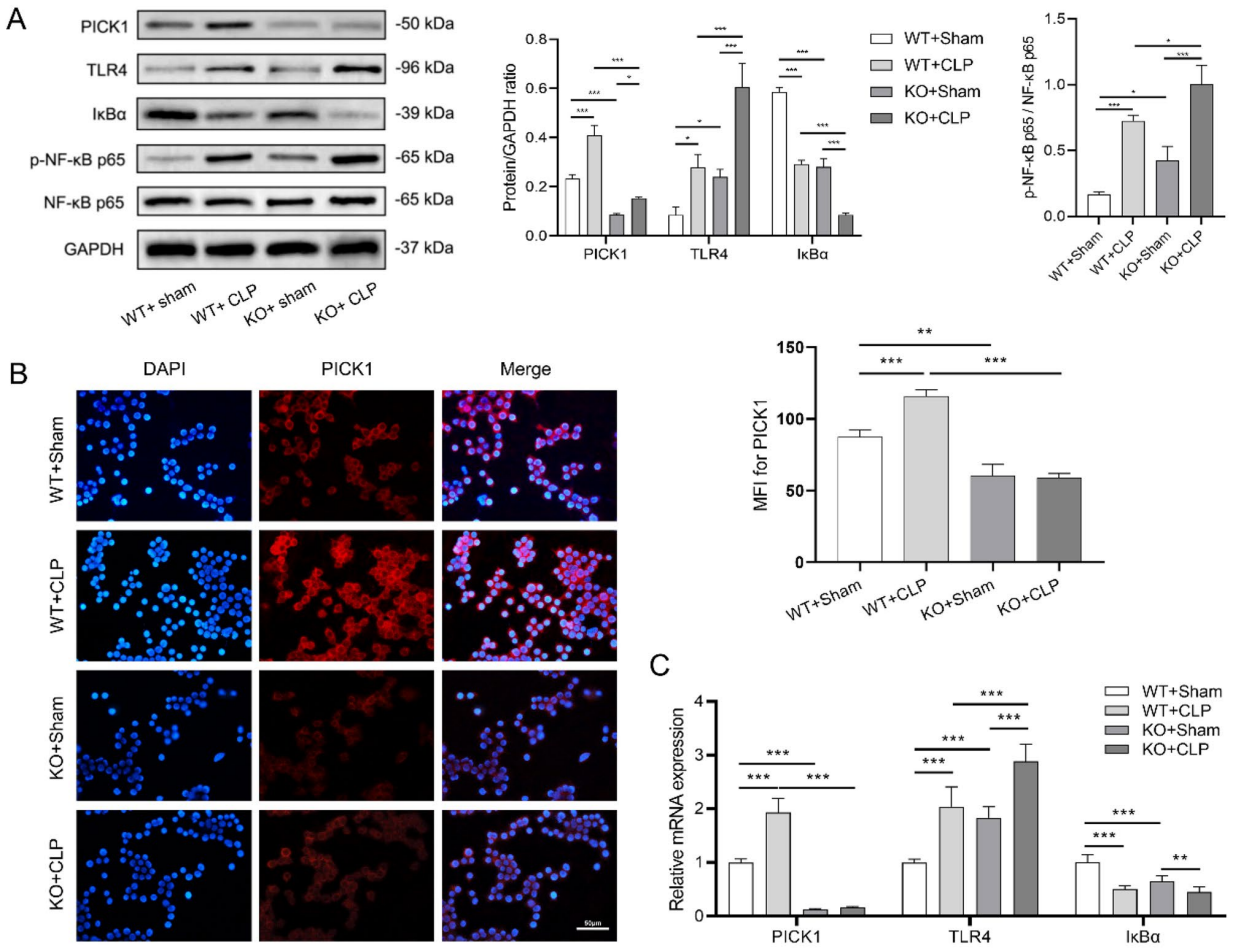
PICK1 knockout activates TLR4/NF-κB signaling pathway in liver macrophages (n = 7). (**A**) The protein expression of PICK1, TLR4, IκBα, and the phosphorylation levels of the NF-κB p65 of each group were detected by western blot; (**B**) PICK1 expression and localization in macrophages detected by immunofluorescence assay; (**C**) The mRNA levels of PICK1, TLR4 and IκBα of each group were detected by RT-PCR. *p < 0.05, **p < 0.01, ***p < 0.001.

The liver macrophages were isolated from murine livers, and the results of western blot revealed that the PICK1 protein expression was significantly higher in the WT + CLP group compared to the WT + sham group (*p* < 0.001). Moreover, the TLR4 protein expression and the NF-κB p65 phosphorylation levels were noticeably higher in the KO groups compared to the WT groups (*p* < 0.05, *p* < 0.001), and that in the CLP groups were significantly increased than those in the sham group (*p* < 0.05, *p* < 0.001), while the trend of protein expression changes of IκBα was opposite to p-NF-κB p65, as it is a suppressor protein of NF-κB (Fig. 5A).

Furthermore, the expression and localization of PICK1 in macrophages were analyzed using the immunofluorescence assay. The findings showed that PICK1 was mostly found in the cytoplasm, and that the WT + CLP group had considerably greater PICK1 MFI levels than the WT + sham group (*p* < 0.001) (Fig. 5B). Additionally, the PCR analysis of macrophages revealed that the level of PICK1 mRNA was significantly higher in the WT + CLP group than in the WT + sham group (*p* < 0.001). Moreover, the KO groups had dramatically higher TLR4 mRNA levels than the WT groups (*p* < 0.001), and the CLP groups had observably higher TLR4 mRNA levels than the sham groups (*p* < 0.001), while the mRNA expression trend of IκBα was found to be opposite to that of TLR4 (Fig. 5C).

**Figure 6:**
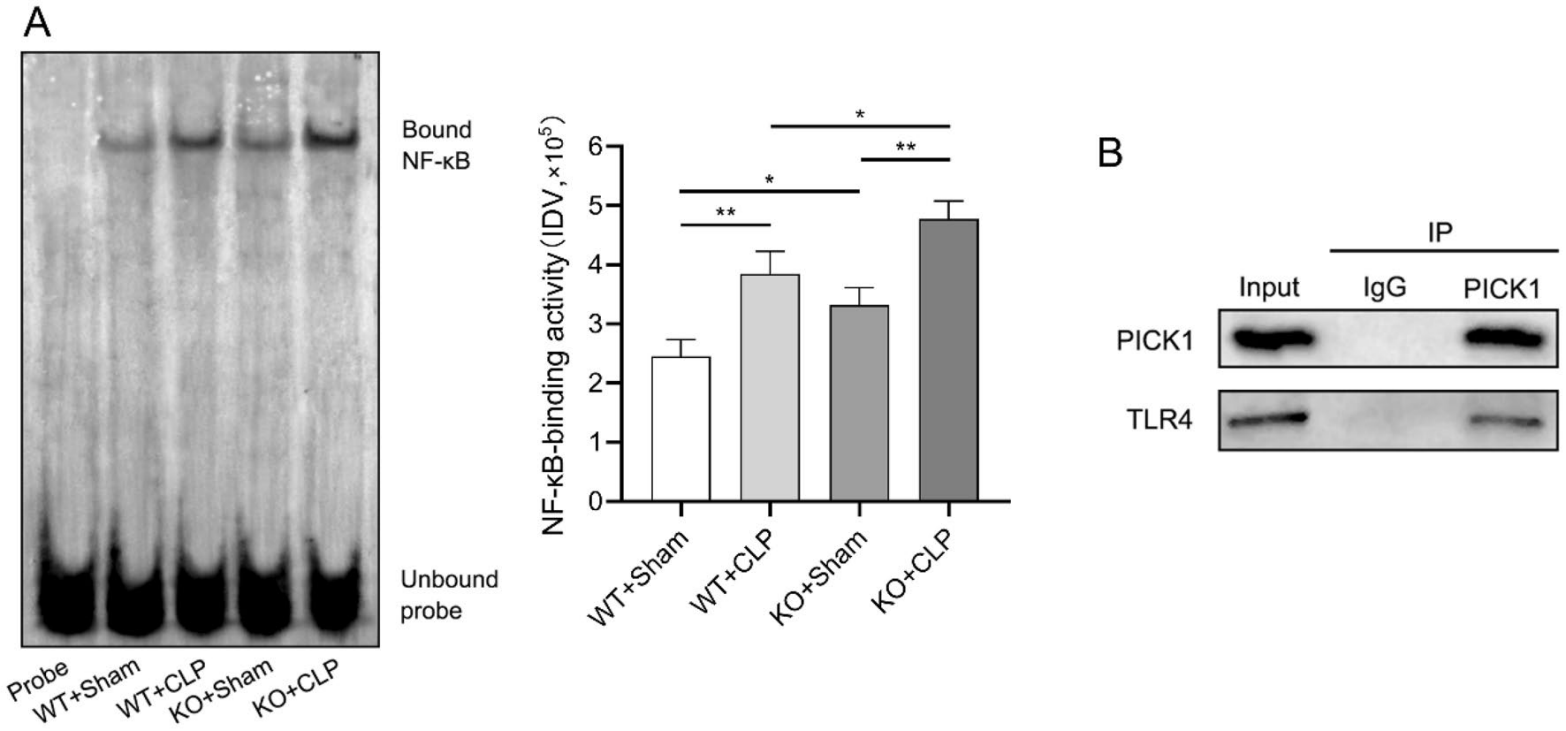
Interaction of PICK1 with NF-κB and TLR4. In liver macrophages, (**A**) the NF-κB activity was examined by EMSA; (**B**) The interaction between PICK1 and TLR4 was detected by co-immunoprecipitation. *p < 0.05, **p < 0.01.

Through EMSA assay, it was found that the NF-κB activity was considerably greater in the KO groups than in the WT groups (*p* < 0.05), and it was notably higher in the CLP groups compared to the sham groups (*p* < 0.01), with the KO + CLP group exhibiting the highest NF-κB activity (Fig. 6A). In addition, the co-immunoprecipitation results revealed that PICK1 and TLR4 could interact with each other (Fig. 6B), suggesting that the PICK1 protein inhibits NF-κB activity via the TLR4 signaling pathway to regulate the release of inflammatory factors, thereby exerting its protective effect on the liver.

## Discussion

Sepsis is an epidemic disease, carrying a high mortality rate due to dysregulation of the body’s inflammatory response, triggered by infectious diseases. Sepsis-induced ALI is a complicated pathophysiological process involving a range of molecular and signaling pathways, including inflammation, apoptosis, and necrosis. Our investigation entailed establishing an inflammatory cell model induced by LPS and a sepsis-induced hepatic injury animal model induced by CLP. The results of this study indicated that the PICK1 protein may play a crucial role in protecting the liver by obstructing NF-B activity through the TLR4 signaling pathway and regulating the release of inflammatory factors.

LPS is a membrane component of Gram-negative bacteria and a crucial substance in the induction of sepsis. It can activate various inflammatory cells, leading to the secretion of various pro-inflammatory mediators and cytokines, making it widely used as an inducer to simulate cellular inflammation^17^. Additionally, the Kupffer cells used in our cell experiments constitute more than 80% of the total macrophages in the body, making them the largest population of macrophages in the body. Kupffer cells reside in the liver sinusoids and are the primary cells responsible for phagocytosis and clearance of circulating toxins. Upon activation by endotoxins, Kupffer cells release a large number of inflammatory factors, ultimately causing liver and multi-organ dysfunction^18–20^. Furthermore, PICK1 is widely distributed in various tissues and organs of the body, particularly in the brain, testes, lungs, liver, kidneys, and muscles, and its expression is significantly increased in peripheral blood monocytes/macrophages of patients with sepsis, showing a correlation with the severity of the disease^21^. Xie et al.^16^ demonstrated that the mRNA level and protein expression of PICK1 in tissues from the ALI mice were significantly reduced, and the inflammatory response in mice was remarkably amplified by PICK1 inhibition, which is consistent with our findings. Specifically, LPS stimulation led to a significant increase in the mRNA level and protein expression of PICK1, and the knockdown of PICK1 resulted in a significant increase in the levels of three inflammatory factors and an increase in cellular apoptosis. In addition, our observations revealed that under LPS stimulation and PICK1 knockdown conditions, both the mRNA and protein expression of TLR4 rose. This finding is in line with previous studies that have reported a significant upregulation of TLR4 mRNA in mice with acute liver failure, and there is a positive correlation between the levels of TNF-α and the expression of TLR4 mRNA in liver tissue^13^. Toll-like receptors are immune recognition receptors, and TLR4 is the most extensively studied member of the TLR family. Following LPS binding via MD-2, TLR4 induces MyD88-dependent signaling from the cell surface, which activates the NF-κB pathway^22^. Consequently, the increased mRNA expression of TLR4 in the liver is likely to induce the inflammatory response of liver tissue to LPS by initiating the transcriptional expression of downstream inflammatory response genes and cytokine release. Moreover, the western blot results of the LPS-induced inflammatory cell model further revealed that the knockdown of PICK1 would increase the expression of TLR4 and NF-κB, thereby elevating the p65 expression. Dong et al. have highlighted the significance of IκBα/p65 in the regulation of LPS-induced inflammation^23^. The LPS-induced inflammatory cell model with PICK1 knockdown exhibited the highest level of apoptosis, and studies have shown that PICK1 confers resistance to apoptosis^24, 25^. The present study developed a mouse model of sepsis-induced liver injury to better explore the potential mechanism of PICK1.

CLP has been shown to more accurately mimic the pathological process of sepsis by inducing a mixed bacterial infection in the peritoneal cavity of mice, thereby leading to acute peritonitis, followed by bacterial entry into the bloodstream and promoting a systemic inflammatory response syndrome^26^. A previous study in a CLP-induced sepsis model demonstrated that PICK1 is associated with sepsis-induced lung injury^27^. This result was supported by the finding of our study, which demonstrated that PICK1 inhibited the TLR4/NF-B pathway activation in the CLP sepsis-induced liver injury mouse model, which protected against sepsis-induced ALI. The TLR4/NF-κB pathway has been implicated in protecting the liver in multiple investigations^28–32^, which is consistent with the findings of our study. By observing the livers of mice, the severity of liver injury in the sepsis-induced liver injury mouse model was found to be highest in mice with PICK1 knockout. The impact of the PICK1 protein on the TLR4 signaling pathway was further investigated by detecting mouse macrophages, which revealed that PICK1 knockout sepsis-induced liver injury mice had the strongest NF-κB activity in sepsis-induced liver injury mice. Previous research has suggested that overexpressed PICK1 could suppress NF-κB activity^16^. Moreover, a previous study illustrated that the PICK-TLR4 complex in microglia was involved in electroacupuncture to prevent neuroinflammation in sepsis-related encephalopathy^33^. Wang et al. have previously demonstrated that TLR4 can form a complex with PICK1 and then be transported to the cell membrane^34^, and the PICK1/TLR4 complex has also been implicated in the regulation of sepsis^35^. Consistent with this, the PICK1 in macrophages was found to interact with TLR4 protein via co-immunoprecipitation in this study. Hence, we postulate that the mechanism underlying the interaction between PICK and TLR4 may involve the PICK1 possesses the capability to engage with TLR4 and reside within the cytoplasm, thereby impeding TLR4 from reaching the membrane and mediating inflammatory responses. In both the Kupffer cell and CLP models, the substantial increase in TLR4 expression subsequent to the knockout of PICK1 suggests that PICK1 potentially mitigates the propagation of inflammatory reactions through its association with TLR4.

In the current investigation, it was found that the knockdown of PICK1 increased the levels of inflammatory factors and apoptosis, and activated the TLR4/NF-κB signaling pathway-related factors in both in vivo and in vitro models. Furthermore, the EMSA revealed an increase in NF-κB activity after PICK1-knockdown, and the co-immunoprecipitation assay indicated an interaction between PICK1 and TLR4. Therefore, this study posits that within sepsis-induced acute liver injury, PICK1 potentially exerts a protective role on the liver by associating with TLR4 to inhibit the release of inflammatory factors and hepatocyte apoptosis. However, the prognosis of PICK1 in sepsis mice remains to be investigated, and we hope to study it in future experiments. In conclusion, this work explored the impact of PICK1 on the regulation of TLR4 and NF-κB expression, as well as the sepsis-induced ALI in CLP mice, providing a research direction for the prevention and treatment of the sepsis-induced ALI.

## Material and methods

### LPS-stimulated inflammatory cell model

To establish an acute inflammation cell model, C57 Kupffer cells (Sciencell, USA) were cultured in RPMI 1640 cell culture medium (Thermo Fisher Scientific, USA). The LPS group was treated with a diluent of 10 g/mL LPS, while the Control group received the treatment of DMSO. Subsequently, the culture plates were incubated at an incubator maintained at a constant 37 °C and 5% CO_2_ for 24 h.

### CLP sepsis-induced liver injury mouse model

Twenty C57BL/6 mice (WT) were purchased from the SPF (Beijing) Biotechnology Co., Ltd, and twenty PICK1 knockout mice (KO) were purchased from the GemPharmatech LLC (Nanjing, China). The mice were fasted 12 h and allowed to drink watered freely before the experiment. The sepsis-induced liver injury model was established through the CLP method as follows: Mice were weighed and anesthetized with an intraperitoneal injection of 10% sodium phenobarbital at an anesthetic dose of 3.5 ml/kg based on mass. A BD 16G needle was used to make a hole in the mesentery of the ligated section of the cecum at 1/2 of the total length contralateral and at a point 1/4 distal to the cecum. Next, a portion of the intestinal contents was extruded to ensure that the perforation was open, after which the cecum was retracted into the abdominal cavity. Subsequently, the abdominal muscles were sutured with silk thread, and corn oil (5 ml/kg) was intraperitoneally injected prior to closing the abdomen. The cecum of the mice in the sham group was only turned after opening the abdominal cavity. Each mouse received a subcutaneous injection of normal saline (3 ml/100 g) and ceftriaxone (30 mg/kg) every 6 h after surgery. The postoperative 10-day survival rates of each group of mice are presented in Supplementary Fig. 1.

After the commencement of the formal experiment, the mice were randomly grouped into the following groups: (1) WT + CLP group (n = 10); (2) WT + sham group (n = 10); (3) KO + CLP group (n = 10); (4) KO + sham group (n = 10). After 24 h post-surgery, mice were sacrificed in each group, and their liver tissues and liver macrophages were collected for following experiment. This study was conducted in accordance with the principles of the Declaration of Helsinki and was approved by the Ethics Committee of Taizhou Hospital of Zhejiang Province Affiliated to Wenzhou Medical University (tzy-2020064), and followed the recommendations in the ARRIVE guidelines.

### Cell transfection

The mouse-specific siRNA against PICK1 genes were purchased from Ribobio (Guangzhou, China). The siRNA sequences were shown as follows: siPICK1: 5′-AAAUACCUGG ACGAUAUAGAG-3′, siCtrl: 5′-UUUCUCCGA ACGUGUCA-3′. Cells were seeded in six-well plates at a density of 1.6 × 10^4^ cells per well with triplicate repetitions. After 24 h, according to the manufacturer's protocols, the cells were transfected with 2 mL transfection mixtures, which included 200 µL Optimem, 5 µL lipofectamine 2000 (Carlsbad, USA), and 5 µL of 20 mM siRNA. The transfection mixture was removed 8 h later, and cells were grown for another 40 h in DMEM/F-12 medium with 5% FBS. The optimal transfection system was used for subsequent experiments.

### Enzyme linked immunosorbent assay (ELISA)

ELISA was performed according to a previous detailed report^36^. After retrieving the supernatant from the inflammatory cells, it was carefully collected following centrifugation at 2000 × g for 20 min. Then, the collected supernatant or standard samples were added to ELISA plates, mixed, and incubated at 37 °C for 40 min, in accordance with the introductions provided by the Westang Biotech Elisa kit (Thermo Fisher Scientific, USA). Following the wash, primary antibodies, enzyme-labeled antibodies, and substrate working solution were added respectively. Finally, after adding the stop reagent, the absorbance was measured at 450 nm using a microplate reader.

### Western blot

Western blot was performed according to a previous detailed report^37^. Protein samples were transferred to PVDF membranes after subjecting them to 10% SDS-PAGE. At room temperature, the membranes were blocked with 5% skim milk for 2 h. Subsequently, they were incubated overnight at 4 °C with the following primary antibodies: PICK1 (1: 1000 dilution), TLR4 (1: 1000 dilution), NF-κB p65 (1: 1000 dilution), IκBα (1: 1000 dilution), p-NF-κB p65 (1: 1000 dilution), cleaved-PARP-1 (1: 1000 dilution), Caspase 1 (1: 1000 dilution), and GAPDH (1: 2500 dilution) in the blocking buffer. On the following day, the membranes were washed with TBST (3 × 10 min) and incubated with the appropriate horseradish peroxidase-conjugated secondary antibodies for 1 h at room temperature. After washing, the protein bands were then detected using the ECL Plus chemiluminescent system, and the image intensity was analyzed with the ImageJ software.

### Real-time quantitative PCR (RT-qPCR)

RT-qPCR was performed according to a previous detailed report^38^. The extraction of total RNA from each group of cells and mouse whole blood was performed using the Trizol reagent, following the manufacturer's protocol. The RNA concentration was determined by measuring the optical density at 260 nm on a spectrophotometer. Reverse transcription was carried out using a PrimeScriptTM RT reagent kit, and 2 μL cDNA was used for the PCR reaction. The PCR products were analyzed via using 1.5% agarose gel electrophoresis and ethidium bromide (EB) staining. Finally, the gels images were analyzed using the Quantity One software. The list of primers used in our paper can be found in Table 1.

**Table 1:** The primers used in Real-Time Quantitative PCR.

Gene	Primer sequences
PICK1	
Forward primer	TCCTGTGTAACGATGGCTTG
Reverse primer	ATGCGCCACTATATGCCTTC
TLR4	
Forward primer	TCCCTGCATAGAGGTAGTTCC
Reverse primer	AGAGGTGGTGTAAGCCATGC
IκBα	
Forward primer	TCGCTCTTGTTGAAATGTGG
Reverse primer	TCATAGGGCAGCTCATCCTC
GAPDH	
Forward primer	TGGAAGGGCTCATGACCACAG
Reverse primer	GGGGTCTGGGATGGAAATTGT

## Isolation of mouse liver macrophages

Mice were sacrificed by cervical dislocation, followed by soaking in 95% alcohol for 3 to 5 min. Then, the abdominal cavity was opened, and the inferior vena cava was severed. Once there was no blood in the liver, the perfusate was replaced with an enzyme solution, the liver was freed and separated using forceps, and the remaining residue was removed with a filter. The cell suspension received 7 mL of PRMI 1640 culture media, and 1 mL of fetal bovine serum was added to a 25 mL culture flask. After mixing, macrophages were cultured until they adherent. Adherent cells were collected by adding EDTA-2Na and centrifuged for 10 min. The cells were diluted to 1.5 × 10^6^ cells/mL after being washed three times with RPMI 1640 medium.

### Hematoxylin and eosin (H&E) staining

The liver of each mouse was fixed in 4% buffered paraformaldehyde, dehydrated with different graded alcohol series, embedded in paraffin and cut into 5 μm sections. The sections were stained with H&E and examined under a Nikon microscope (Thermo Fisher Scientific, USA), and the images were analyzed by image Pro-Plus 7200 software.

### Immunofluorescence staining

Immunofluorescence staining was performed according to a previous detailed report^39^. In brief, cells were seeded in a 24-well cell culture plate and treated with different treatments for 24 h. Then, the cells were fixed with 4% paraformaldehyde for 15 min, permeabilized with 0.5% Triton X-100, and blocked with 5% bovine serum albumin for 20 min. Finally, the cells were incubated with a primary antibody targeting PICK1 (Abcam, UK, 1: 200) at 4 °C overnight and incubated with an Alexa Fluor 488-labeled Goat Anti-Rabbit IgG (Abcam, UK, 1: 1000). The nucleus was stained with 4’,6-diamidino-2-phenylindole (DAPI), and fluorescence was captured on a Zeiss Axio Imager.Z2 microscope system.

### Electrophoretic mobility shift assay (EMSA)

The nuclear proteins of livers macrophages were extracted, bound, and reacted with universal κB double-stranded oligonucleotide probe sequences P1: 5′-AGCTTCAGAGGGG ACTTTCCGAGAGG-3′; P2: 3′-TCGAAGTCTCCCCTG AAAGGCTCTCC-5′. The 0.5 × TBE solution was used as the membrane transfer solution to transfer the probes, proteins, and probe-protein complexes in the EMSA gel (Thermo Fisher Scientific, USA) to a nylon membrane. The nylon membrane was cross-linked under 254-nm ultraviolet light in UV cross-linking equipment, and the biotin-labeled probes were detected by a chemiluminescent method followed by X-ray film exposure. The gray-scale value of each band was measured using Image J software.

### Co-Immunoprecipitation (Co-IP)

Cell lysates were incubated with antibodies at 4 °C overnight, followed by incubation with protein A/G magnetic beads (Thermo Scientific, USA). Subsequently, the beads were collected and subjected to IB. The antibodies used for co-IP were anti-PICK1 (Abcam, UK), anti-TLR4 (Abcam, UK) and anti-lgG (Abcam, UK).

### Statistical analysis

The data were expressed as mean ± standard deviation (x ± s), and the data from each experiment were analyzed and calculated 3 times using SPSS 18.0 data analysis software. To examine the differences of continuous variables of four groups, a one-way analysis of variance (ANOVA) was conducted followed by Tukey’s post-hoc test. *p* < 0.05 was considered statistically significant. *p < 0.05, **p < 0.01, ***p < 0.001.

## Ethical approval and informed consent

This study was performed in line with the principles of the Declaration of Helsinki. This study was approved by the Ethics Committee of Taizhou Hospital of Zhejiang Province Affiliated to Wenzhou Medical University (tzy-2020064), and followed the recommendations in the ARRIVE guidelines.

## Supplementary Information

Supplementary Figure 1.

## Data Availability

The datasets generated during the current study are available from the corresponding author on reasonable request.

## References

[1] Singer M (2016). The third international consensus definitions for sepsis and septic shock (Sepsis-3). JAMA.

[2] Fleischmann C (2016). Assessment of global incidence and mortality of hospital-treated Sepsis. Current estimates and limitations. Am. J. Respirat. Crit. Care Med..

[3] Yan J, Li S, Li S (2014). The role of the liver in sepsis. Int. Rev. Immunol..

[4] Lelubre C, Vincent J (2018). Mechanisms and treatment of organ failure in sepsis. Nat. Rev. Nephrol..

[5] Strnad P, Tacke F, Koch A, Trautwein C (2017). Liver - guardian, modifier and target of sepsis. Nat. Rev. Gastroenterol. Hepatol..

[6] Jensen K, Noes-Holt G, Sørensen A, Madsen K (2021). A novel peripheral action of PICK1 inhibition in inflammatory pain. Front. Cell. Neurosci..

[7] Sørensen A, Rombach J, Gether U, Madsen K (2022). The scaffold protein PICK1 as a target in chronic pain. Cells.

[8] Li Y, Zhang N, Wang Y, Shen Y, Wang Y (2016). Multiple faces of protein interacting with C kinase 1 (PICK1): Structure, function, and diseases. Neurochem. Int..

[9] Qian M (2018). PICK1 deficiency exacerbates sepsis-associated acute lung injury and impairs glutathione synthesis via reduction of xCT. Free Rad. Biol. Med..

[10] Dou Q, Tong H, Yang Y, Zhang H, Gan H (2021). PICK1 deficiency exacerbates sepsis-associated acute kidney injury. BioMed. Res. Int..

[11] Ramachandran G (2014). Gram-positive and gram-negative bacterial toxins in sepsis: a brief review. Virulence.

[12] Takayashiki T (2004). Increased expression of toll-like receptor 4 enhances endotoxin-induced hepatic failure in partially hepatectomized mice. J. Hepatol..

[13] Jiang W, Sun R, Wei H, Tian Z (2005). Toll-like receptor 3 ligand attenuates LPS-induced liver injury by down-regulation of toll-like receptor 4 expression on macrophages. Proc. Natl. Acad. Sci. U. S. A..

[14] Chen X, Sun Z, Zhang H, Wang L (2021). kCorrelation of impaired NF-B activation in sepsis-induced acute lung injury (ALI) in diabetic rats. J. Healthc. Eng..

[15] Li S (2021). Dental pulp stem cell-derived exosomes alleviate cerebral ischaemia-reperfusion injury through suppressing inflammatory response. Cell Prolif..

[16] Xie J (2016). PICK1 confers anti-inflammatory effects in acute liver injury via suppressing M1 macrophage polarization. Biochimie.

[17] Dang CP, Leelahavanichkul A (2020). Over-expression of miR-223 induces M2 macrophage through glycolysis alteration and attenuates LPS-induced sepsis mouse model, the cell-based therapy in sepsis. PLoS One.

[18] Selvaraj V (2015). Inhibition of MAP kinase/NF-kB mediated signaling and attenuation of lipopolysaccharide induced severe sepsis by cerium oxide nanoparticles. Biomaterials.

[19] Wu XQ (2016). Telomerase reverse transcriptase acts in a feedback loop with NF-kappaB pathway to regulate macrophage polarization in alcoholic liver disease. Sci. Rep..

[20] Cho HI (2015). beta-Caryophyllene alleviates D-galactosamine and lipopolysaccharide-induced hepatic injury through suppression of the TLR4 and RAGE signaling pathways. Eur. J. Pharmacol..

[21] Qian M (2018). PICK1 deficiency exacerbates sepsis-associated acute lung injury and impairs glutathione synthesis via reduction of xCT. Free Radic. Biol. Med..

[22] Kagan JC, Medzhitov R (2006). Phosphoinositide-mediated adaptor recruitment controls Toll-like receptor signaling. Cell.

[23] Dong N (2020). Astragalus polysaccharides alleviates LPS-induced inflammation via the NF-κB/MAPK signaling pathway. J. Cell. Physiol..

[24] Wang W (2007). Mitochondrial anchoring of PKCalpha by PICK1 confers resistance to etoposide-induced apoptosis. Apoptosis Int. J. Program. Cell Death.

[25] Xiao N (2009). PICK1 deficiency causes male infertility in mice by disrupting acrosome formation. J. Clin. Invest..

[26] Rittirsch D, Huber-Lang M, Flierl M, Ward P (2009). Immunodesign of experimental sepsis by cecal ligation and puncture. Nat. Protoc..

[27] Mo Y (2018). PICK1 deficiency induces autophagy dysfunction via lysosomal impairment and amplifies sepsis-induced acute lung injury. Med. Inflam..

[28] Li J (2021). Novel roles of lipopolysaccharide and TLR4/NF-κB signaling pathway in inflammatory response to liver injury in Budd–Chiari syndrome. World J. Gastrointest. Surg..

[29] Lu J (2021). Resveratrol modulates Toxoplasma gondii infection induced liver injury by intervening in the HMGB1/TLR4/NF-κB signaling pathway. Eur. J. Pharmacol..

[30] Wang M (2021). Alprostadil alleviates liver injury in septic rats via TLR4/NF-κB pathway. Eur. Rev. Med. Pharmacol. Sci..

[31] Wei S (2019). RIP3 deficiency alleviates liver fibrosis by inhibiting ROCK1-TLR4-NF-κB pathway in macrophages. FASEB J. Off. Publ. Fed. Am. Soc. Exp. Biol..

[32] Weng J, Wang X, Xu B, Li W (2021). Augmenter of liver regeneration ameliorates ischemia-reperfusion injury in steatotic liver via inhibition of the TLR4/NF-κB pathway. Exp. Therapeut. Med..

[33] Mo Y (2021). Electroacupuncture prevents LPS- induced neuroinflammation via upregulation of PICK-TLR4 complexes in the microglia of hippocampus. Brain Res. Bull..

[34] Wang D (2010). Ras-related protein Rab10 facilitates TLR4 signaling by promoting replenishment of TLR4 onto the plasma membrane. Proc. Natl. Acad. Sci. U. S. A..

[35] Wang L (2021). The PICK1/TLR4 complex on microglia is involved in the regulation of LPS-induced sepsis-associated encephalopathy. Int. Immunopharmacol..

[36] Ye X, Xiang F, Hu Y (2023). Gambogic acid affects high glucose-induced apoptosis and inflammation of retinal endothelial cells through the NOX4/NLRP3 pathway. Ann. Transl. Med..

[37] Jin YH, Min JS, Kwon S (2023). Cardamonin as a p38 MAPK signaling pathway activator inhibits human coronavirus OC43 infection in human lung cells. Nutrients.

[38] Livak KJ, Schmittgen TD (2001). Analysis of relative gene expression data using real-time quantitative PCR and the 2(-Delta Delta C(T)) method. Methods.

[39] Wang Y (2021). Quercetin alleviates acute kidney injury by inhibiting ferroptosis. J. Adv. Res..

