# Supplementary figures and images for: Knockdown of protein interacting with C α kinase 1 aggravates sepsis-induced acute liver injury by regulating the TLR4/NF-κB pathway
Source: Sci Rep. 2023 Jul 24;13:11913. doi: 10.1038/s41598-023-38852-w (PMC10366226; doi:10.1038/s41598-023-38852-w)

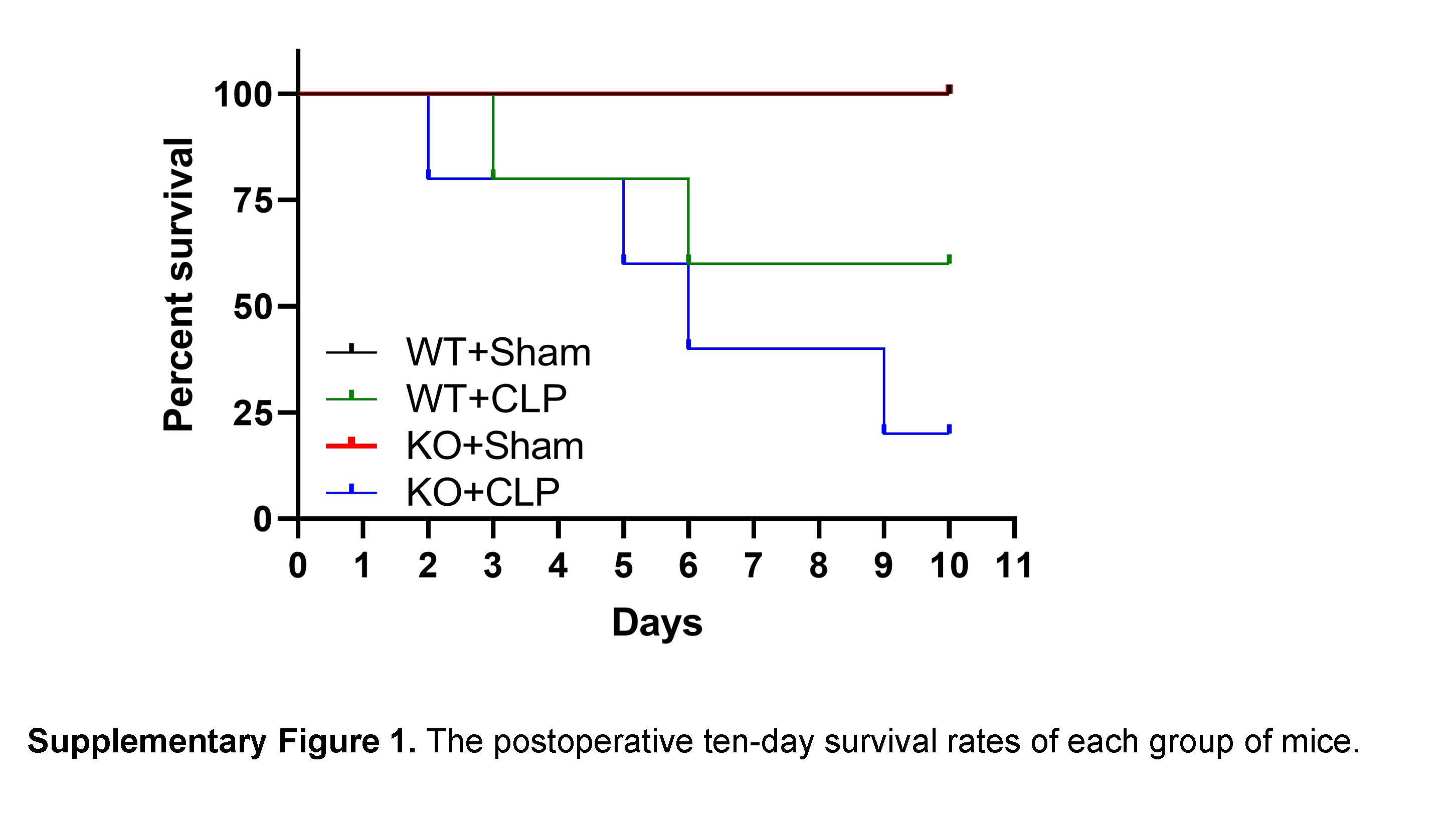

Supplement: Supplementary file 1 — Supplementary Figure 1. [file 41598_2023_38852_MOESM1_ESM.tif]
